# Antibiotics from predatory bacteria

**DOI:** 10.3762/bjoc.12.58

**Published:** 2016-03-30

**Authors:** Juliane Korp, María S Vela Gurovic, Markus Nett

**Affiliations:** 1 Leibniz Institute for Natural Product Research and Infection Biology – Hans-Knöll-Institute, Beutenbergstr. 11, 07745 Jena, Germany; 2 Centro de Recursos Naturales Renovables de la Zona Semiárida (CERZOS) -CONICET- Carrindanga Km 11, Bahía Blanca 8000, Argentina; 3 Department of Biochemical and Chemical Engineering, Technical Biology, Technical University Dortmund, Emil-Figge-Strasse 66, 44227 Dortmund, Germany

**Keywords:** antibiotics, genome mining, *Herpetosiphon*, myxobacteria, predation

## Abstract

Bacteria, which prey on other microorganisms, are commonly found in the environment. While some of these organisms act as solitary hunters, others band together in large consortia before they attack their prey. Anecdotal reports suggest that bacteria practicing such a wolfpack strategy utilize antibiotics as predatory weapons. Consistent with this hypothesis, genome sequencing revealed that these micropredators possess impressive capacities for natural product biosynthesis. Here, we will present the results from recent chemical investigations of this bacterial group, compare the biosynthetic potential with that of non-predatory bacteria and discuss the link between predation and secondary metabolism.

## Introduction

Microorganisms are major contributors to primary biomass production and nutrient cycling in nature. The composition of a microbial community shapes an ecosystem, but is also responsive to biotic and environmental cues. Predation is among the ecological forces, which drive the diversity and dynamics of microbial consortia [1–3]. While protozoa and nematodes are widely known as bacterivores [4–5], the existence of predatory prokaryotes is often neglected despite the abundance of the latter and their early occurrence in the history of life, likely preceding eukaryotic predators [6–9].

Predatory behavior is in fact not uncommon for bacteria. It can be observed in many different species, which are found in the actinobacteria (e.g., *Agromyces ramosus*) [10], the chloroflexi (e.g., *Herpetosiphon* spp.) [11–12], the proteobacteria (e.g., *Bdellovibrio bacteriovorus*, *Myxococcus xanthus*, *Ensifer adhaerens*, *Cupriavidus necator*, *Lysobacter* spp.) [13–17], the bacteroidetes (e.g., *Saprospira grandis*, *Tenacibaculum* spp.) [18–19], and even in the cyanobacteria (e.g., *Vampirovibrio chlorellavorus*) [20]. Depending on their feeding behavior, that is, whether or not their diet relies exclusively on prey consumption, these bacteria have been classified as obligate or facultative predators [6]. While obligate predators can only survive by consuming other bacteria, facultative predators readily switch to a saprophytic lifestyle in the absence of appropriate preys [21]. Another division of predatory bacteria is based on their hunting strategies [22]. Epibiotic predation involves attachment to the outer surface of the prey, which is then followed by a degradation of the prey’s cell wall and assimilation of cell components through specialized structures [23]. Other predatory bacteria are known to directly penetrate the prey cell in a process called diacytosis [24–25] or to selectively invade the periplasm of Gram-negative bacteria [26]. The corresponding behaviors are referred to as endobiotic and periplasmic predation, respectively [22]. Another strategy, which is called group or ‘wolfpack’ predation, is only practiced by facultative predators. A prerequisite for this collaborative type of hunting is a quorum of predatory cells, which pool hydrolytic enzymes, proteases or nucleases in order to lyse and feed on nearby prey [22].

Group predation occurs predominantly in bacteria, which also display social swarming behavior, gliding motility and sophisticated communication systems. Illustrative examples include the myxobacteria, as well as *Lysobacter* and *Herpetosiphon* species [6,27–29]. Members of these taxa are further characterized by their large genome sizes and their striking potential for the production of structurally diverse natural products with antimicrobial activities [12,30–35]. For many years, it has been speculated whether antibiotic biosynthesis is functionally linked to the predatory lifestyle of these organisms [27,36]. In this review, we will address this unresolved question both from a genomic perspective and on the basis of chemical investigations. Terrestrial myxobacteria and the genus *Herpetosiphon* will be in the focus of our analysis, whereas *Lysobacter* spp., which have just been the subject of a comparative metabolomics study [37], are not covered. For information on marine myxobacteria, readers are referred to the review article by König et al. in this Thematic Series [38].

## Review

### Biology and biosynthetic potential of myxobacteria

Myxobacteria are ubiquitous soil bacteria with a complex life cycle, which involves the coordinated differentiation from individual cells into multicellular fruiting bodies under starvation conditions [39–40]. Furthermore, myxobacteria are distinguished by their unique gliding motility allowing a rapid swarming dispersal [41], which likely also benefits their predation strategy. Considering their highly sophisticated developmental program and their manifold social interactions, it is not surprising that fruiting myxobacteria are among the prokaryotes with the largest genomes. Their genomes typically range from 9 up to 15 Mbp in size and contain between 7,285 (*Myxococcus fulvus* HW-1) and 11,599 (*Sorangium cellulosum* So0157-2) protein-coding sequences (Table 1) [42–47]. In comparison, the genome of the standard laboratory bacterium *Escherichia coli* comprises only 4.6 Mbp of DNA [48]. With a single exception, all myxobacterial genomes that have been sequenced to date consist of a single circular chromosome and feature no plasmids [42–47,49–50]. To evaluate the biosynthetic capabilities of the myxobacterial strains listed in Table 1, their genome sequences were scanned for the presence of putative secondary metabolite gene clusters using the publicly available online tool antiSMASH 3.0 [51]. This analysis revealed that all strains possess extraordinary capacities for natural product assembly. Interestingly, however, the number of biosynthetic loci is not linearly correlated with the genome size. The largest number of secondary metabolite gene clusters was found in *Corallococcus coralloides* DSM 2259 and not in the two *Sorangium cellulosum* strains, although the latter feature significantly larger genomes (Table 1). When the number of detected loci is related to the genome size, it becomes obvious that the Cystobacterineae strains consistently possess more biosynthesis gene clusters per Mbp of DNA than the analyzed Sorangiineae and that they also devote a larger percentage of their total nucleotides to natural product biosynthesis. Noteworthy in this context, the genera *Myxococcus* and *Corallococcus*, on the one hand, as well as the genus *Sorangium*, on the other, represent different nutritional types among the myxobacteria. Only the former are bacteriolytic and attack other microorganisms, whereas the latter live as cellulose degraders [36,52–54]. Although mere numbers of biosynthesis gene clusters provide no information about the identity or biological role of the associated natural products, we note that predatory myxobacteria possess a higher density of secondary metabolite gene clusters in their genomes than their non-predatory relatives.

**Table 1:** Taxonomic assignment, nutrition, genomic and biosynthetic features of myxobacterial strains.

	*Myxococcus fulvus* HW-1	*Myxococcus xanthus* DK1622	*Corallococcus coralloides* DSM 2259	*Myxococcus stipitatus* DSM 14675	*Sorangium cellulosum* So ce56	*Sorangium cellulosum* So0157-2

Suborder	Cystobacterineae	Cystobacterineae	Cystobacterineae	Cystobacterineae	Sorangiineae	Sorangiineae
Family	Myxococcaceae	Myxococcaceae	Myxococcaceae	Myxococcaceae	Polyangiaceae	Polyangiaceae
Nutrition	saprotrophic predatory	saprotrophic predatory	saprotrophic predatory	saprotrophic predatory	saprotrophic, cellulolytic	saprotrophic, cellulolytic
Genome size [bp]	9,003,593	9,139,763	10,080,619	10,350,586	13,033,779	14,782,125
Protein-coding sequences	7,285	7,388	8,033	8,043	9,367	11,599
GenBank accession no.	CP002830	CP000113	CP003389	CP004025	AM746676	CP003969
Reference	[42]	[43]	[44]	[45]	[46]	[47]
# of biosynthesis gene clusters^a^	25	24	36	29	31	34
# of biosynthesis gene clusters per Mbp	2.78	2.63	3.57	2.80	2.38	2.30
Combined length of biosynthesis clusters [bp]^a^	1,147,796	1,329,413	1,571,607	1,672,930	1,199,901	1,450,537
Genome portion devoted to biosynthesis [%]	12.75	14.55	15.59	16.16	9.21	9.81

^a^Numbers and size of biosynthesis loci were determined using antiSMASH [50].

But are these clusters indicators for predatory behavior? – To answer this question, we will take a closer look at their metabolic products using *Myxococcus xanthus* DK1622 as an example. This strain, a model organism for the analysis of myxobacterial fruiting body development and motility, feeds on a number of different soil bacteria upon direct contact by a mechanism called predatory rippling [14,55]. Although the biology of *M. xanthus* DK1622 had been thoroughly investigated for decades, the bacterium did not come into the focus of natural product chemists until the sequencing of its genome. Bioinformatic analysis of the DK1622 chromosome with antiSMASH indicated the presence of 24 gene clusters, which are involved in the secondary metabolism (Table 2).

**Table 2:** Biosynthetic gene clusters in the genome of *M. xanthus* DK1622 and their predicted or known products.^a^

No.	Cluster location	Type	Actual or predicted product	Estimated size [kb]

1	MXAN_0889-MXAN_0906	terpene	carotenoid	21.0
2	MXAN_1276-MXAN_1312	NRPS	dipeptide	46.3
3	MXAN_1508-MXAN_1543	other	unknown	44.4
4	MXAN_1588-MXAN_1624	NRPS	hexapeptide	64.6
5	MXAN_2782-MXAN_2814	NRPS/PKS (type I)	unknown	51.8
6	MXAN_2847-MXAN_2864	lantipeptide	class II lantipeptide	23.3
7	MXAN_3447-MXAN_3479	PKS (type I)	unknown	46.7
8	MXAN_3551-MXAN_3559	bacteriocin	bacteriocin	10.9
9	MXAN_3602-MXAN_3658	NRPS/PKS (type I) + NRPS	lipopeptide + **myxochelin**	168.4
10	MXAN_3763-MXAN_3797	NRPS/PKS (type I)	**myxoprincomide**	82.8
11	MXAN_3917-MXAN_3957	*trans*-AT-PKS/NRPS	**myxovirescin**	109.6
12	MXAN_3986-MXAN_4020	NRPS/PKS (type I)	lipopeptide	70.3
13	MXAN_4057-MXAN_4100	PKS (type I)/NRPS	**myxochromide**	69.0
14	MXAN_4156-MXAN_4166	bacteriocin	bacteriocin	11.7
15	MXAN_4271-MXAN_4312	PKS (type I)/NRPS	**DKxanthene**	76.9
16	MXAN_4384-MXAN_4402	NRPS/PKS (type I)	unknown	48.2
17	MXAN_4404-MXAN_4438	NRPS/PKS (type I)	lipopeptide	70.0
18	MXAN_4508-MXAN_4549	NRPS/PKS (type I)	**myxalamide**	92.7
19	MXAN_4545-MXAN_4561	lantipeptide	lantipeptide	26.2
20	MXAN_4578-MXAN_4618	NRPS	lipopeptide	79.4
21	MXAN_4951-MXAN_4960	bacteriocin	bacteriocin	10.8
22	MXAN_6241-MXAN_6257	terpene	geosmin	22.2
23	MXAN_6377-MXAN_6414	lantipeptide/ladderane/ PKS (type II)	unknown	41.1
24	MXAN_6618-MXAN_6659	PKS (type III)	alkylresorcinol	41.1

^a^All predictions are according to [50], except for the assignment of the myxochelin gene cluster.

**Figure 1:**
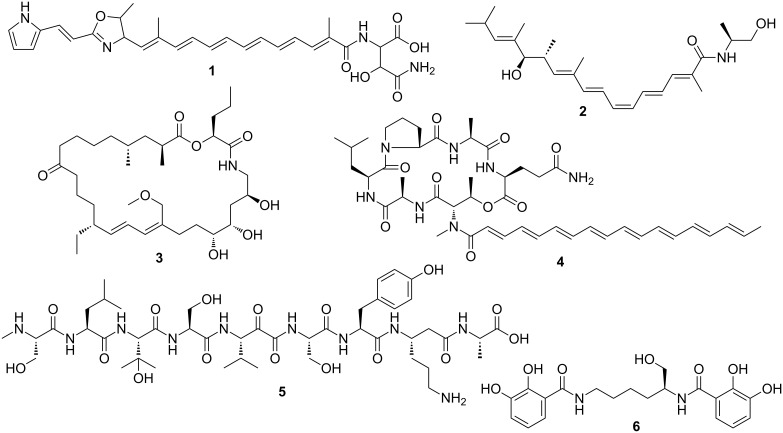
Natural products isolated from *M. xanthus* DK1622. DKxanthene-534 (**1**); myxalamid B (**2**); myxovirescin A_1_ (**3**); myxochromide A_3_ (**4**); myxoprincomide (**5**); myxochelin A (**6**).

Until now, six loci have been associated with isolated natural products on the basis of biosynthetic precedence and extensive metabolome analyses (Figure 1) [56–57]. While some of the retrieved compounds from *M. xanthus* DK1622 are also known from different myxobacterial species, as exemplified by the myxochelins [58–60] and myxochromides [61–62], others were initially discovered in this strain, such as the myxoprincomides [57] and the DKxanthenes [63].

The known secondary metabolites of *M. xanthus* DK1622 show a wide range of biological activities and can hence be expected to fulfill different ecological functions. The yellow DKxanthenes, for instance, play a crucial role in spore maturation during fruiting body formation [63]. They were also shown to possess antioxidative properties and might thus confer resistance towards oxidative stress [63]. Structurally, the DKxanthenes harbor a hydrophilic asparagine moiety attached to a hydrophobic polyene chain bearing an additional oxazoline and pyrrol ring system. Their production seems to be universal among *Myxococcus* strains and several derivatives varying in their polyene chain length as well as extent of methyl branching have been identified [63–65]. The myxochromides represent another pigment family commonly encountered in myxobacteria [61,65–66]. While their chemistry and biosynthesis have been thoroughly explored [62,67], the biological function of these cyclic depsipeptides is still not clear. In contrast, the myxochelins primarily serve as siderophores for *M. xanthus* DK1622, as evidenced by their iron-responsive production and complexing properties [58–59]. Recent studies also unveiled specific enzymatic targets for these natural products [60,68], which are not due to their iron affinity [69]. Myxalamids [70–73] and myxovirescins [74–79] are distinguished by their potent antimicrobial activities. The former are inhibitors of electron transport in the respiratory chain. They were shown to block the electron flow at complex I of mitochondria (NADH:ubiquinone oxidoreductase) in a competitive manner, but do not act on bacterial complex I [71–72]. This explains why the myxalamids are mainly active against fungi [71].

The myxovirescins comprise a family of closely related antibiotics featuring a distinctive 28-membered macrolide ring. First discovered by Rosenberg et al. in *M. xanthus* TA [74], the myxovirescins were later also reported from other myxobacterial isolates, including strain DK1622 [75–79]. Myxovirescins are excreted during late exponential and early stationary growth phase and display strong inhibitory activities on growing bacterial cells, even when applied at concentrations less than 5 µg/mL. Toxicity against eukaryotic cells was not observed [74,80]. Myxovirescin A_1_ was found to be particularly effective against enterobacteria with a minimal inhibitory concentration (MIC) of 1 µg/mL [75]. Its mode of action was deduced after genetic characterization of myxovirescin-resistant *E. coli* mutants [81]. The antibiotic interferes with cell-wall biosynthesis by inhibiting a novel target, i.e., the type II signal peptidase LspA, which is involved in the maturation of lipoproteins required for murein biosynthesis [81].

Myxovirescin A_1_ (also known as antibiotic TA) and its derivatives seem to be of particular importance for the predatory lifestyle of *M. xanthus* DK1622. Gene deletion experiments demonstrated that a loss of myxovirescin biosynthesis significantly affects the ability of the myxobacterium to kill actively growing *E. coli* cells [82]. Furthermore, myxovirescin-resistant *E. coli* strains were shown to be largely resistant against predation by DK1622, demonstrating for the first time a clear link between antibiotic production and predation. However, myxovirescins cannot be considered as universal predatory weapons for *M. xanthus* DK1622, as the macrolides have no effects on the Gram-positive prey bacterium *Micrococcus luteus* [82]. It remains unclear whether as yet unidentified antibiotics from the DK1622 metabolome complement the bioactivity of myxovirescins and, thereby, expand the prey spectrum. Alternatively, it is possible that the killing of *M. luteus* involves a different predation strategy (e.g., attack with hydrolytic enzymes).

In any case, the coordinate production of antibiotics, such as myxovirescin A, requires a tight regulatory network in predatory myxobacteria [80]. This is also reflected in the genome of *M. xanthus* DK1622, which features an unusual high duplication frequency of genes encoding regulatory proteins like serine-threonine kinases and enhancer binding proteins (EBPs) [43]. EBPs are regulatory proteins influencing the transcription by binding to a specific enhancer-like element (ELE) sequence located in close vicinity to the corresponding promoter in a δ54 dependent manner [83]. Two EBPs of *M. xanthus* DK1622, namely HsfA and MXAN4899, have recently been identified as transcriptional regulators of secondary metabolism via DNA–protein pull-down assays [84]. Knock-out studies revealed that both EBPs are necessary for the formation of intact fruiting body and sporulation. DKxanthene biosynthesis was strongly influenced by HsfA and MXAN4899, respectively, which is in good agreement with the biological function of this compound class [63]. Furthermore, the two EBPs were linked to the regulation of the myxovirescin pathway and motility. While HsfA acted as a repressor of the myxovirescin production, MXAN4899 could exert enhancing or inhibitory effects depending on the nutrition status of the myxobacterium. The findings of this study attested a complex regulatory network to *M. xanthus* DK1622, in which development, predation, and motility are clearly connected to secondary metabolism [84].

Lastly, it should be mentioned that genomic data might also provide the explanation for the predatory behavior of some myxobacteria. Nutritional studies had shown that *M. xanthus* cannot be grown in the absence of branched-chain amino acids [85]. Consistent with these results, the genome of strain DK1622 lacks *ilvC* and *ilvD* genes, which are required for the biosynthesis of these amino acids. It was hence speculated that predation might compensate for this deficiency [43]. Analysis of the other myxobacterial genomes now lends support to this assumption. We found the absence of *ilvC* and *ilvD* to be a consistent trait in the bacteriolytic *Myxococcus* and *Corallococcus* strains, whereas the genomes of the cellulolytic *Sorangium* strains harbor well-conserved homologs of both genes.

### Antibiotics from myxobacteria with a possible role in predation

The following listing highlights few selected antibiotics from myxobacteria in the context of predation. For a comprehensive overview of bioactive compounds from myxobacteria and their modes of action, the reader is referred to the excellent review articles by König et al. [33] and Müller et al. [86].

**Figure 2:**
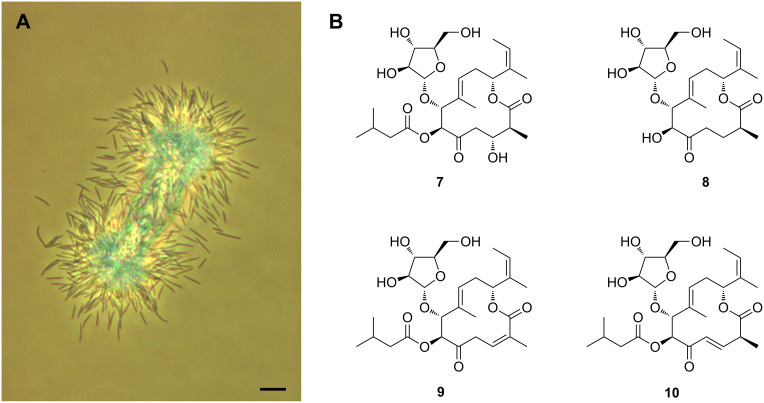
Vegetative cells of *P. fallax* HKI 727 under a phase-contrast microscope (K. Martin, unpublished). Bar is 10 μm (A). Structures of gulmirecin A (**7**), gulmirecin B (**8**), disciformycin A (**9**), and disciformycin B (**10**) (B).

**Gulmirecins:** The gulmirecins were found in a culture broth of the predatory myxobacterium *Pyxidicoccus fallax* HKI 727 (Figure 2) [87]. Their discovery is an illustrative example on how new antibiotics can be retrieved from predatory bacteria. The isolation of predatory bacteria from soil is typically achieved by means of baiting techniques. For this, a pea-sized sample is placed on a nutrient-poor agar medium, that was previously inoculated with potential prey microbes [88–90]. These organisms serve as attractants that will allow the enrichment of any predators present in the soil sample. Baiting techniques have proven to be particularly useful for the recovery of bacteriolytic myxobacteria, as swarming and fruiting body formation facilitate the separation from other microorganisms [91]. If the isolation procedure is repeated with varying "food organisms", it becomes possible to select for myxobacteria that can be distinguished by their preference for certain prey bacteria. This approach was also used during the isolation of strain HKI 727, which readily consumed the prey bacterium *Bacillus subtilis*, but not *Escherichia coli*. Further tests revealed that *P. fallax* HKI 727 exhibits a prey range that is restricted to Gram-positive bacteria. Culture extracts of strain HKI 727 showed a consistent antimicrobial profile, i.e., they were highly active against Gram-positive bacteria. Bioactivity-guided fractionation then led to the identification of the gulmirecins as the active principles [87].

Chemically, the gulmirecins form a novel class of antibiotics together with the disciformycins [92], which were discovered in a different *P. fallax* strain upon a large-scale screening. The distinctive 12-membered macrolide scaffold in these natural products features an arabinose moiety (Figure 2), which is only rarely observed in bacterial polyketides. The main difference between gulmirecins A and B is the presence or absence of an isovalerate substituent. Comparison with the bioactivity data of the disciformycins suggests that the isovalerate motive is important for the antibacterial activity. Due to their potent effects against human pathogenic staphylococci as well as negligible toxicity, gulmirecins A and disciformycin B have become promising candidate compounds for the design of new antibiotics [93–94]. Since the gene loci that are involved in their biosyntheses have been identified [87,92], it might even be possible to genetically engineer further derivatives in the future. The close correlation between the activity profile of the gulmirecins and the prey range of strain HKI 727 further suggests that isolation procedures for predatory bacteria can be directed in order to obtain strains producing antibiotics against specific pathogens.

**Figure 3:**
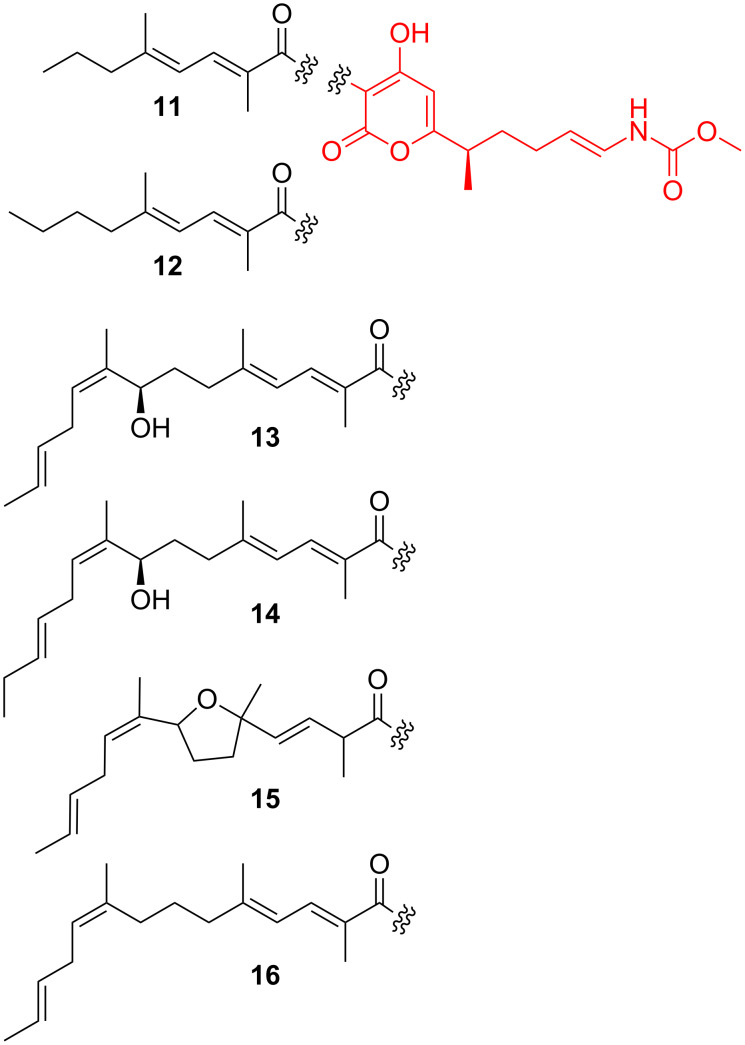
Structures of myxopyronins A (**11**) and B (**12**), corallopyronins A (**13**), B (**14**) and C (**15**), as well as precorallopyronin A (**16**).

**Myxopyronins and corallopyronins:** The myxopyronins were first reported in 1983 from a culture supernatant of *Myxococcus fulvus* Mxf50 [95–96]. Later, the structurally related corallopyronins were found in different strains of *Corallococcus coralloides* [97–99]. Myxopyronins and corallopyronins share a common scaffold composed of a central pyrone ring carrying two flexible side chains (Figure 3). Structural variability manifests in the so-called western side chain, which ranges from 10 (myxopyronin A) up to 18 carbon atoms (corallopyronin B). In contrast, the eastern chain is conserved among all members and features a terminal methyl carbamate moiety. Differences in the architectures of the respective biosynthetic assembly lines were recently shown to account for the diverging frameworks of the western chain [100–102].

Myxopyronins and corallopyronins turned out to be highly active against Gram-positive bacteria with MIC values between 0.1 and 1.0 µg/mL for *Staphylococcus aureus*, whereas their inhibitory effects on Gram-negative strains are in general much weaker. Gram-negative bacteria of the genus *Wolbachia*, which have emerged as a new target for filariasis control, constitute a significant exception [103]. Already in the 1980s, incorporation studies with labeled precursors revealed the inhibition of prokaryotic RNA polymerase (RNAP) as mode of action for myxopyronins and corallopyronins [95,97]. Later on, mutagenesis experiments as well as binding studies indicated that the antibiotics interact with the RNAP switch region [104–105], which acts as a hinge mediating conformational changes during transcription [106]. During early stages of transcriptional initiation, the RNAP clamp possesses an opened form in order to allow binding of the promoter DNA to the active-center cleft. At late transcriptional initiation and elongation, the clamp changes into a closed position to retain the DNA inside the active-center cleft. After binding to the switch region, myxopyronins and corallopyronins prevent the opening of the clamp [104–105].

Prey bacteria that develop resistance against corallopyronin, e.g., due to a *rpoB* mutation, also become resistant towards predation by *C. coralloides* [82]. It is thus likely that corallopyronin is produced by myxobacteria to facilitate feeding on other bacteria.

**Figure 4:**
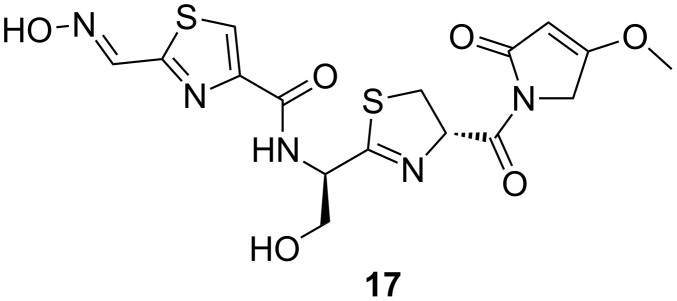
Structure of althiomycin (**17**).

**Althiomycin:** The antibiotic althiomycin (Figure 4) had been initially discovered in cultures of *Streptomyces althioticus* [107], before it was also reported from strains of *Myxococcus virescens, M. xanthus, and Cystobacter fuscus* [108]. The pentapeptide is broadly active against Gram-positive as well as Gram-negative bacteria and was shown to selectively inhibit bacterial protein synthesis. Its specific site of inhibition is the 50S subunit of the ribosome, where althiomycin interferes with the peptidyl transferase reaction [109–110]. The althiomycin biosynthetic gene cluster was recently identified in *M. xanthus* DK897 by a combination of retrobiosynthetic analysis and gene inactivation [111]. Two open reading frames (ORFs) encoding for a nonribosomal peptide synthetase (NRPS) and a NRPS/polyketide synthase (PKS) hybrid were found to be involved in the assembly of the core structure. Furthermore, the cluster included four additional ORFs that have specific roles in tailoring reactions and drug resistance [111].

Unlike the ubiquitous DKxanthenes or myxochelins, althiomycin is only produced by a few members of the species *M. xanthus* [65]. For instance, the model strain DK1622 lacks the althiomycin biosynthesis genes and is even sensitive against this antibiotic [111]. In a comprehensive chemical analysis of 98 different *M. xanthus* strains, althiomycin was never observed together with myxovirescins [65]. It is hence very tempting to speculate that the predatory weapon myxovirescin could have been replaced by another potent antibiotic. Considering the dispersal of althiomycin biosynthesis genes in many taxonomically unrelated bacteria [112], it appears possible that some myxobacteria acquired the respective locus via horizontal gene transfer.

**Figure 5:**
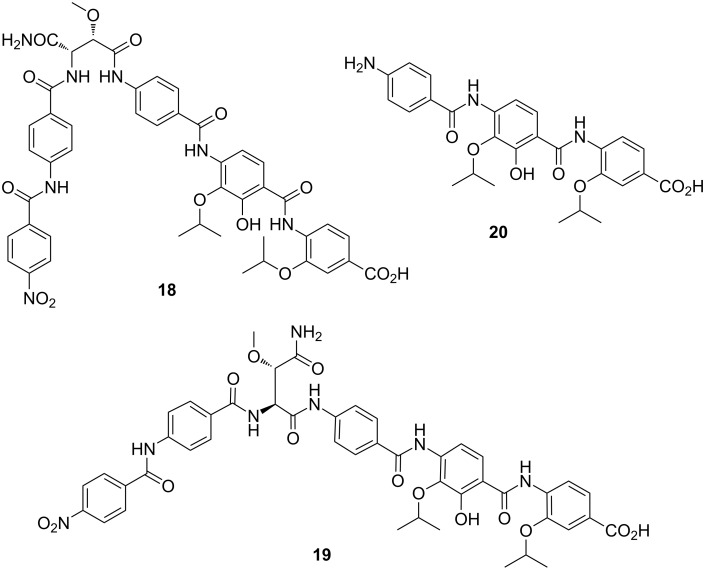
Structures of cystobactamids 919-1 (**18**), 919-2 (**19**), and 507 (**20**).

**Cystobactamids:** The cystobactamids were recently isolated from a *Cystobacter* sp. and represent a novel class of NRPS-derived antimicrobial peptides [113]. Cystobactamids 919-1 and 919-2 (Figure 5) display an unusual aromatic scaffold composed of *p-*nitrobenzoic acid and four *p*-aminobenzoic acid (PABA)-derived moieties. The latter vary in their oxidation and substitution pattern, which may even comprise rare isopropoxy groups. The two unmodified PABA residues in compounds 919-1 and 919-2 are connected via an *iso*-β-methoxyasparagine or a β-methoxyasparagine unit, respectively. In contrast, the tripeptidic cystobactamid 507 seems to be either a biosynthetic byproduct or a degradation fragment of its larger congeners. All cystobactamids lack antifungal and cytotoxic properties, but they exhibit significant antibacterial activities. Especially derivative 919-2 (**19**) possesses strong inhibitory effects on the growth of Gram-positive and Gram-negative bacteria. Susceptible bacteria include *Acinetobacter baumannii*, which is a frequent inhabitant of soil, but has received even more attention as a causative agent of hard-to-treat nosocomial infections [113].

Analysis of the cystobactamid biosynthesis gene cluster led to the identification of a gene encoding a putative resistance factor. This discovery was the starting point for resolving the molecular target of these antibiotics. Subsequent assays confirmed that the cystobactamids act as bacterial DNA gyrase inhibitors [113]. Whether or not the cystobactamids are involved in predation has not been investigated yet. Their potent activity at nanomolar concentrations against a broad range of bacteria would undoubtedly make them excellent molecules for hunting down prey.

### Biology and biosynthetic potential of *Herpetosiphon* spp.

Taxonomically, the genus *Herpetosiphon* belongs to the class Chloroflexi within the homonymous phylum. Members of this phylum are metabolically highly diverse, including dehalorespiring anaerobes besides aerobic CO-oxidizing thermophiles, chlorophototrophs and chemoheterotrophs [114–116]. Characteristic features in the class Chloroflexi comprise a filamentous morphology and gliding motility. The associated bacteria stain Gram-negative, albeit lacking a lipopolysaccharide-containing outer membrane [117], and they typically grow phototrophically under anoxic conditions [118]. In stark contrast to its relatives, *Herpetosiphon* is not capable of photosynthesis. It has been proposed that the genus diverged from the major lineage upon loss of its photosystem and has shifted to a saprophytic, facultative predatory lifestyle [114]. *Herpetosiphon* spp. seem to be widely distributed in soil and freshwater environments [119], where they attack and digest a multitude of bacteria [11]. Akin to myxobacteria, they are assumed to practice group predation [6]. Actually, the genus only includes two validly described species, namely *H. aurantiacus* and *H. geysericola*. The genome of *H. aurantiacus* 114-95^T^, which is the type species of the entire genus, was fully sequenced and annotated [12]. Furthermore, a draft genome sequence of *H. geysericola* has recently become available [120]. The circular chromosomes of the two *Herpetosiphon* strains are of comparable size, i.e., 6.35 and 6.14 Mbp, whereas their phototrophic relatives have smaller replicons that range from 4.68 to 5.80 Mbp [12]. It thus appears as if there has been an enlargement of the predator’s genomes. Some of the expansion that is evident results from the acquisition of genes involved in secondary metabolism [121]. While the potential of *Chloroflexus* and *Roseiflexus* spp. for the production of natural products is negligible [122], the *Herpetosiphon* genomes contain a significant number of biosynthetic loci (Table 3). Unlike actinomycete genomes, which are particularly rich in polyketide pathways [123], the *Herpetosiphon* chromosomes were found to be dominated by NRPS or mixed NRPS/PKS clusters. This situation is hence quite similar to myxobacteria [56]. An unexpected finding, however, was the discovery of an enediyne PKS gene in *H. aurantiacus* 114-95^T^. Enediynes are highly potent antibiotics, causing DNA-strand scissions. Although an impressive number of 87 enediyne clusters could be identified in sequencing projects over the past years, comparatively few loci were retrieved from microbes outside the actinobacteria [124]. This suggests an event of horizontal gene transfer (HGT) in *H. aurantiacus* 114-95^T^. Analysis of a large NRPS/PKS cluster in the same strain yielded even more compelling evidence for HGT. Not only is the respective cluster enclosed by a number of transposon fragments, it also features an above-average G+C content of ≈66% (the genome standard is 50.9%) as well as significant G+C shifts in its border regions [12]. The observation that HGT is in part responsible for the accumulation of biosynthesis genes is again reminiscent of the predatory myxobacteria [43].

**Table 3:** Taxonomic assignment, nutrition, genomic and biosynthetic features of Chloroflexi bacteria.

	*Herpetosiphon aurantiacus* 114-95^T^	*Herpetosiphon geysericola* GC-42	*Chloroflexus aurantiacus* J-10-fl	*Chloroflexus aggregans* DSM 9485	*Roseiflexus castenholzii* DSM 13941	*Roseiflexus* sp. RS-1

Order	Herpetosiphonales	Herpetosiphonales	Chloroflexales	Chloroflexales	Chloroflexales	Chloroflexales
Nutrition	saprotrophic predatory	saprotrophic predatory	phototrophic	phototrophic	phototrophic	phototrophic
Chromosome size [bp]	6,346,587	6,140,412 (draft)	5,258,541	4,684,931	5,723,298	5,801,598
Protein-coding sequences	5,577	4,688	3,853	3,679	4,492	4,639
GenBank accession no.	CP000875	NZ_ LGKP00000000	CP000909	CP001337	CP000804	CP000686
Reference	[12]	[120]	[122]	GenBank	GenBank	GenBank
# of biosynthesis gene clusters^a^	14	9	2	4	4	4
# of biosynthesis gene clusters per Mbp	2.21	1.47	0.38	0.43	0.70	0.69
Combined length of biosynthesis clusters [bp]^a^	821,829	300,554	42,182	42,170	117,838	117,958
Genome portion devoted to biosynthesis [%]	12.95	4.89	0.80	0.90	2.06	2.03

^a^Numbers and size of biosynthesis loci were determined using antiSMASH [50].

### Natural products from *Herpetosiphon* spp.

**Figure 6:**
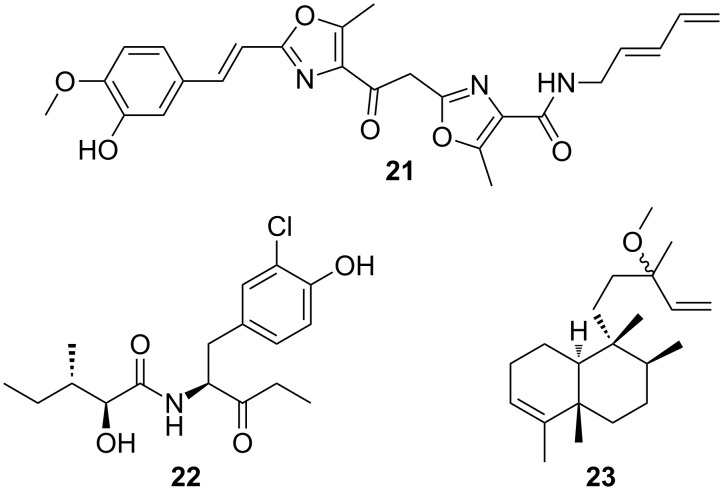
Structures of natural products isolated from *Herpetosiphon* spp.: siphonazole (**21**); auriculamide (**22**); (+)-*O*-methylkolavelool (**23**).

While myxobacteria are already known as a promising source for natural product research [31–32], the genus *Herpetosiphon* has been almost completely ignored in the field. It is therefore no surprise that to date only three classes of secondary metabolites have been reported from this genus (Figure 6). Siphonazole and its *O*-methyl derivative were the first natural products to be isolated from a *Herpetosiphon* strain [125]. The two compounds exhibit an unusual molecular architecture featuring two oxazole rings connected by a two-carbon tether and a terminal 2,4-pentadienylamine moiety. Feeding experiments as well as biosynthetic reasoning indicated that the siphonazoles originate from a mixed PKS/NRPS pathway [125]. Both bisoxazoles were found to possess anticancer properties, but they lack antimicrobial activities [126]. In case the siphonazoles should contribute to the predatory behavior of the producing strain, they must exert more subtle effects than those described for myxovirescins and gulmirecins.

Efforts to identify secondary metabolites from the *H. aurantiacus* type strain 114-95^T^ led to the discovery of auriculamide (**22**) [127]. This natural product is composed of a 2-hydroxy-3-methylvalerate and a 2-amino-1-(3-chloro-4-hydroxyphenyl)pentan-3-one residue. A retrobiosynthetic analysis allowed the assignment of the gene cluster, which is responsible for the production of auriculamide. According to the current biosynthetic model, the scaffold of auriculamide is assembled on an NRPS/PKS enzyme complex. A decarboxylation reaction was proposed to shorten the off-loaded carboxylic acid and to give rise to the unusual end group of the natural product [127]. Whether auriculamide possesses antibiotic properties is still open. The low fermentation yield prevented biological testing of the isolated compound.

More recently, the terpenome of *H. aurantiacus* 114-95^T^ received some attention. Researchers found two genes in the chromosome, the enzymatic products of which exhibited high sequence similarity to proteins that are responsible for the biosynthesis of tuberculosinol and isotuberculosinol in *Mycobacterium tuberculosis* [128]. Following in vitro studies of the two *Herpetosiphon* enzymes as well as a reconstitution of the entire associated pathway, (+)-*O*-methylkolavelool was identified as a metabolic product. Subsequent GC–MS analyses confirmed that this previously unknown diterpene is actually produced by the predatory bacterium [128].

From genomic data, it is evident that the genus *Herpetosiphon* harbors a significant potential for the biosynthesis of natural products. The fact that no antibiotics have been described from this bacterium yet is in our opinion most likely due to a lack of adequate studies. Similar to other neglected producer organisms [129], the genus *Herpetosiphon* can be expected to yield many previously unknown natural products.

## Conclusion

Bacteria practicing group predation possess comparatively large replicons with an average size of 6.0 to 6.3 Mbp in case of *Lysobacter* and *Herpetosiphon* spp., or 9.6 Mbp in case of *Myxococcus* and *Corallococcus* spp. [12,37,49]. Although the genome size must not necessarily exceed the most closely related non-predatory species, as shown for the myxobacteria, a distinctive feature between predatory and non-predatory strains is the density of biosynthetic loci. In other words, families of genes encoding the production of secondary metabolites were consistently found to be overrepresented in the genomes of predatory bacteria. This observation may reflect a need for specialized molecules that coordinate swarm formation or mediate prey killing. The assumption that the extended biosynthetic capacities are due to the predatory lifestyle can, however, not be verified, because only a small fraction of the corresponding secondary metabolomes have been explored. Even in case of model organisms, which were subject of extensive chemical investigations, such as *M. xanthus* DK1622, the products of most biosynthetic pathways await their discovery. On the other hand, there is now strong evidence that compounds, such as the myxovirescins or corallopyronins, are used by their producers to enable feeding on certain prey bacteria [82]. The loss of these antibiotics or, alternatively, a resistance development of the prey organism could not be compensated by the predator and always resulted in a restricted prey spectrum [82]. Regarding the available information on the chemistry of predatory myxobacteria, it seems likely that every strain has the potential to produce at least a single class of natural products with potent antibacterial activity. The high recovery rate of myxovirescins and corallopyronins in strains of *Myxococcus xanthus* and *Corallococcus coralloides* [65,100] suggests a correlation between taxonomy and secondary metabolism. The discovery of the structurally related gulmirecins and disciformycins in different strains of *Pyxidicoccus fallax* [87,92] further supports the idea of species-specific antibiotics. Is the analysis of new *M. xanthus* isolates hence futile in terms of antibiotic discovery? – The observation of *M. xanthus* strains producing althiomycin instead of myxovirescins indicates the opposite, although the chance for retrieving antibiotics other than myxovirescins from this species might be low [65]. However, it should not be ignored that the activity profile of the identified antibiotics from *M. xanthus* does not cover the entire prey spectrum of this predator.

In summary, predatory bacteria are a promising source to find antibiotics, as these compounds confer a clear advantage to feed on prey organisms. New compound classes can most likely be expected from hardly studied genera and species. Also, it seems advisable to consider the prey preference of a bacterial hunter when searching for antibiotics that are active against selected pathogens.
